# Study on the Structure-Luminescence Relationship and Anti-Counterfeiting Application of (Ca,Sr)-Al-O Composite Fluorescent Materials

**DOI:** 10.3390/nano15181446

**Published:** 2025-09-19

**Authors:** Jianhui Lv, Jigang Wang, Yuansheng Qi, Jindi Hu, Haiming Li, Chuanming Wang, Xiaohan Cheng, Deyu Pan, Zhenjun Li, Junming Li

**Affiliations:** 1 Beijing Key Laboratory of Printing and Packaging Materials and Technology, Beijing Institute of Graphic Communication, Beijing 102600, China; 17854211937@163.com (J.L.); di13087073292@163.com (J.H.); 17363387595@163.com (H.L.); w_chuanming@163.com (C.W.); c1595631742@163.com (X.C.); 18717155359@163.com (D.P.); 2 National Center for Nanoscience and Technology, CAS Key Laboratory of Nanophotonic Materials and Devices (Preparatory), Beijing 100190, China; 3 The GBA Research Innovation Institute for Nanotechnology, Guangzhou 510700, China; 4 Beijing Key Laboratory for Sensors, Beijing Information Science & Technology University, Beijing 100192, China; li@bistu.edu.cn

**Keywords:** long-lasting luminescence, (Ca,Sr)_3_Al_2_O_6_ host, composite trap engineering, Eu^2+^, Nd^3+^, dual-mode anti-counterfeiting, screen printing, QR code, ink

## Abstract

A novel long-lasting luminescent composite material based on the (Ca,Sr)-Al-O system was synthesized using a solution combustion method. (Ca,Sr)_3_Al_2_O_6_ is the primary phase, with SrAl_2_O_4_ as a controllable secondary phase. Compared to conventional single-phase SrAl_2_O_4_ phosphors, the introduction of a calcium-rich hexaaluminate matrix creates additional defects and a specific trap distribution at the composite interface, significantly improving carrier storage and release efficiency. Eu^2+^ + Nd^3+^ synergistic doping enables precise control of the trap depth and number. Under 365 nm excitation, Eu^2+^ emission is located at ~515 nm, with Nd^3+^ acting as an effective trap center. Under optimal firing conditions at 700 °C (Eu^2+^ = 0.02, Nd^3+^ = 0.003), the afterglow lifetime exceeds 30 s. Furthermore, The (Ca,Sr)_3_Al_2_O_6_ host stabilizes the lattice and optimizes defect states, while synergizing with the SrAl_2_O_4_ secondary phase to improve the afterglow performance. This composite phosphor exhibits excellent dual-mode anti-counterfeiting properties: long-lasting green emission under 365 nm excitation and transient blue-violet emission under 254 nm excitation. Based on this, a screen-printing ink was prepared using the phosphor and ethanol + PVB, enabling high-resolution QR code printing. Pattern recognition and code verification can be performed both in the UV on and off states, demonstrating its great potential in high-security anti-counterfeiting applications. Compared to traditional single-phase SrAl_2_O_4_ systems, this study for the first time constructed a composite trap engineering of the (Ca,Sr)_3_Al_2_O_6_ primary phase and the SrAl_2_O_4_ secondary phase, achieving the integration of dual-mode anti-counterfeiting functionality with a high-resolution QR code fluorescent ink.

## 1. Introduction

Long Persistent Luminescence (LPL) materials have attracted widespread attention due to their ability to store excitation energy during luminescence and continuously release photons after the external stimulus is removed [1,2]. Due to their high brightness, long lifetime, and good chemical stability, LPL phosphors have been widely used in security identification, optical data storage, anti-counterfeiting inks [3,4,5], bioimaging, and laser technology. Among the many LPL systems, rare earth ion-doped aluminates have attracted much attention due to their stable crystal structure, low toxicity, and efficient energy storage capacity. Among them, SrAl_2_O_4_: Eu^2+^, Nd^3+^/Dy^3+^ is considered a typical green LPL phosphor, exhibiting strong emission caused by the Eu^2+^ 4f^6^5d^1^ → 4f^7^ transition at ≈515 nm [6,7,8], with an afterglow time of up to several seconds. However, single-phase SrAl_2_O_4_ systems still face three major challenges:

(i) the difficulty in precisely controlling trap density and energy distribution, which limits the persistence of afterglow;
(ii) the insufficient tunability of emission properties under multi-wavelength excitation; and
(iii) the poor lattice stability under high-temperature conditions, which restricts their application in high-performance functional inks

To overcome these problems, designing multiphase composite LPL systems has become an effective strategy. Compared with single-phase SrAl_2_O_4_, the introduction of calcium-rich hexaaluminate (Ca,Sr)_3_Al_2_O_6_ as the main host phase has two major advantages:

(i) Interface defect engineering: By regulating the lattice mismatch between (Ca,Sr)_3_Al_2_O_6_ and SrAl_2_O_4_, additional electron traps are formed, thereby improving energy storage capacity;
(ii) Enhanced structural stability: The cubic (Ca,Sr)_3_Al_2_O_6_ matrix suppresses grain boundary collapse and maintains trap integrity during high-temperature synthesis.

The synergistic effect of these two aspects significantly improves the long-lasting performance of the composite system, making it have higher brightness, longer lifetime, and better tunability than traditional SrAl_2_O_4_-based phosphor. In this study, we synthesized a novel (Ca,Sr)-Al-O composite LPL material via a solution combustion method [9,10,11,12,13,14]. (Ca,Sr)_3_Al_2_O_6_ serves as the primary phase, with SrAl_2_O_4_ serving as a controllable secondary phase [15,16,17]. By systematically adjusting the Eu^2+^/Nd^3+^ doping concentration, we achieved precise control over the number and depth of traps. Under optimized conditions (Eu^2+^ = 0.02, Nd^3+^ = 0.003), the material exhibited strong green emission at ≈515 nm under 365 nm excitation, with an afterglow lifetime exceeding 30 s.

Notably, this composite system exhibits unique dual-mode anti-counterfeiting properties: Under 365 nm excitation, the material produces a strong green emission with a long afterglow; Under 254 nm excitation, it exhibits transient blue-violet emission.

Based on this, we used Eu^2+^, Nd^3+^ co-doped composite LPL phosphors and ethanol/PVB to formulate anti-counterfeiting inks, and fabricated high-resolution QR codes and fluorescent anti-counterfeiting patterns using screen printing technology. The patterns exhibited blue-violet and green emission under 254 nm and 365 nm UV light, respectively. The resulting QR code pattern also exhibited green emission under 365 nm UV light, and could still be identified by afterglow after the light was turned off. Furthermore, with the help of mobile phone cameras or industrial machine-readable equipment, dual visual and machine-readable verification could be achieved, significantly improving anti-counterfeiting security. This study is the first to propose a dual-mode long-afterglow material based on a (Ca,Sr)_3_Al_2_O_6_ primary phase-SrAl_2_O_4_ secondary phase composite trap engineering, combining high brightness, color tunability, and high-resolution QR code printing, demonstrating its broad application potential in high-value product anti-counterfeiting, bill security, industrial tracking, and other fields.

## 2. Experimental Process

### 2.1. Experimental Reparation

A series of (Ca,Sr)_3_Al_2_O_6_-SrAl2O4: Eu^2+^, Nd^3+^ composite long-lasting fluorescent materials were synthesized by the solution combustion method [10,11,12,13,14]. The raw materials used were CaCO_3_ (purity 99.99%), SrCO_3_ (purity 99.99%), Al_2_O_3_ (purity 99.99%), Eu_2_O_3_ (purity 99.99%), Nd_2_O_3_ (purity 99.99%), and the combustion aids urea (purity 99.99%) and nitric acid (purity 80%), all purchased from Tianjin Chemical Reagent Factory(Tianjin, China). All reagents were used directly in the experiments without further purification.

### 2.2. Material Preparation Process

First, CaCO_3_, SrCO_3_, Al_2_O_3_, Eu_2_O_3_ and Nd_2_O_3_ were dissolved in an appropriate amount of nitric acid to prepare a nitrate precursor solution, the concentrations of which are shown in Table 1:

The nitrate solutions were then mixed in a stoichiometric ratio of Ca:Sr:Al = 1.97:2.03:4 to produce a transparent, homogeneous solution (all solutions were prepared fresh). (Ca,Sr)_3_Al_2_O_6_ was maintained as the primary phase [15], while a SrAl_2_O_4_ secondary phase was permitted to coexist. After identifying the Eu^2+^-doped sample with the best optical performance, the Nd^3+^ doping concentration was adjusted. To investigate the impact of doping on performance, two concentration gradients of Eu^2+^ and Nd^3+^ were designed:

Eu^2+^: 0.01, 0.02, 0.03, 0.05, 0.08

Nd^3+^: 0.001, 0.002, 0.003, 0.004, 0.005, 0.008

Note that the concentration of the nitrate precursor solution is different from the final solid-state molar ratio of Eu^2+^ and Nd^3+^ in the phosphor. The final doping ratios x(Eu^2+^) and y(Nd^3+^) are defined relative to the total molar amount of alkaline earth ions.

To promote the formation of Eu^2+^, we intentionally added urea to the precursor mixture. During heating, the crucible was covered with an alumina lid, leaving a slight gap between the lid and the crucible. This allowed the release of gases such as NH_3_, CO, and CO_2_ during combustion, while also creating a localized low oxygen partial pressure environment within the crucible, suppressing high-temperature reoxidation of Eu^2+^. This temporary protective effect effectively lowered the local oxygen partial pressure, indirectly promoting the in-situ reduction of Eu^3+^ to Eu^2+^, thereby stabilizing the Eu^2+^ in the crystal lattice.

All combustion reactions were performed in a preheated muffle furnace at 700 °C under ambient air without any controlled atmosphere, graphite covering, or sacrificial carbon. The local reduction of Eu^3+^ to Eu^2+^ was achieved in situ by the urea-assisted combustion process, where decomposition of urea generates reducing gases (CO, NH_3_, H_2_) that partially lower the local oxygen partial pressure.

The homogeneous precursor solution containing 1 mmol of alkali metal elements was added to urea (1.1 g) as the fuel and reducing agent, and stirred continuously until completely clear. The solution was poured into a porcelain crucible and placed in a muffle furnace preheated to 700 °C. A self-propagating combustion reaction proceeded rapidly (approximately 9–13 min), producing a bulky, white, porous solid powder. After cooling to room temperature, the powder was ground to obtain samples with varying doping concentrations. To investigate the effect of temperature on the crystalline phase and luminescence properties [17], the optimal Eu^2+^-doped sample was synthesized repeatedly at temperatures of 500, 600, 700, 800, and 900 °C. Previous studies have shown that an appropriate calcination temperature is crucial for ensuring crystal integrity and uniform trap distribution. The resulting product was used for subsequent XRD, SEM, EDS, XPS, PL, and afterglow performance testing [15,16,17,18].

**Figure 1:**
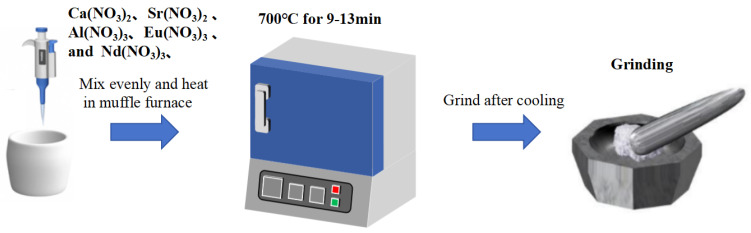
Experimental preparation process of phosphor.

The experimental preparation process is shown in Figure 1:

### 2.3. Characterization of Materials

The crystal structure of the synthesized samples was determined using a powder X-ray diffractometer (D/max 2200 PC, Rigaku Corporation, Tokyo, Japan) with Cu Kα radiation (λ = 1.54 Å), recording diffraction peaks within the range of 10° to 70°. Photoluminescence (PL) and photoluminescence excitation (PLE) spectra were measured using a fluorescence spectrometer (F-4700, Hitachi High-Tech, Tokyo, Japan). Ultraviolet diffuse reflectance absorption spectra were measured using a UV-3600 UV-Vis-NIR spectrometer (Shimadzu Corporation, Kyoto, Japan). The fluorescence lifetime and afterglow decay curves of the phosphors were measured using a transient steady-state fluorescence spectrometer (FLS1000, Edinburgh Instruments, Livingston, UK). The morphology of the phosphors was observed using a scanning electron microscope (Quanta 250 FEG, Thermo Fisher Scientific, Waltham, MA, USA). The elemental composition and content were measured using a scanning electron microscope equipped with an energy-dispersive X-ray spectrometer (Apreo C, Thermo Fisher Scientific, Waltham, MA, USA).

## 3. Test Result Analysis

### 3.1. Phase Composition and Structural Evolution

**Figure 2:**
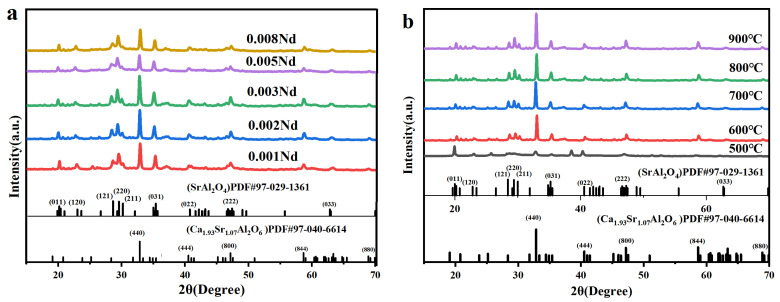

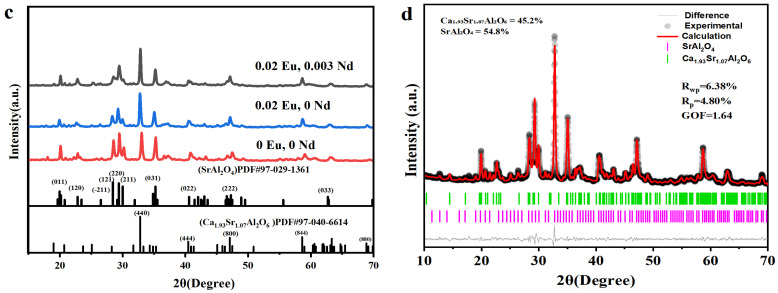
(**a**) XRD patterns of (Ca,Sr)-Al-O: 0.02 Eu^2+^, y Nd^3+^ phosphors at different Nd^3+^ doping concentrations (y = 0.001, 0.002, 0.003, 0.005, 0.008) compared with a standard card; (**b**) XRD patterns of (Ca,Sr)-Al-O: 0.02 Eu^2+^, 0.003 Nd^3+^ phosphors at different calcination temperatures (500 °C, 600 °C, 700 °C, 800 °C, 900 °C) compared with a standard card; (**c**) XRD pattern comparison of (Ca,Sr)-Al-O phosphors under different ion doping conditions, including undoped samples, Eu^2+^ single doping (0.02), and Eu^2+^-Nd^3+^ phosphors. Co-doped (Eu^2+^ = 0.02, Nd^3+^ = 0.003) and compared with a standard card. (**d**) Rietveld refinement results and phase composition analysis of the (Ca,Sr)_3_Al_2_O_6_-SrAl_2_O_4_: Eu^2+^, Nd^3+^ composite phosphors.

Powder X-ray diffraction (XRD) was used to analyze the phase composition of Eu^2+^, Nd^3+^ co-doped (Ca,Sr)-Al-O phosphors synthesized via a solution combustion method (Figure 2) [19,20,21,22]. All samples exhibited clear diffraction peaks, indicating high crystallinity within the investigated doping and temperature ranges. The primary reflection peak matches the cubic (Ca,Sr)_3_Al_2_O_6_ phase (PDF#97-040-6614), while additional peaks at ~28°, ~32°, and ~33° correspond to the monoclinic SrAl_2_O_4_ phase (PDF#97-029-1361), confirming the coexistence of the primary (Ca,Sr)_3_Al_2_O_6_ phase and a controlled SrAl_2_O_4_ secondary phase [23].

Unlike conventional SrAl_2_O_4_-based LPL phosphors, the introduction of a calcium-rich hexaaluminate phase significantly alters the microstructural evolution of the (Ca,Sr)_3_Al_2_O_6_/SrAl_2_O_4_ interface. These interfacial mismatches create additional defect sites and shallow traps, which are expected to improve carrier storage and release kinetics, thereby enhancing persistent luminescence performance. Figure 2b further illustrates the effect of calcination temperature (500–900 °C) on phase composition. As the synthesis temperature increases from 500 °C to 700 °C, the diffraction peaks gradually become sharper and the full width at half maximum (FWHM) decreases, indicating improved crystallinity and optimized phase distribution. At 700 °C, the ratio of primary to secondary phases is maximized, thus balancing structural stability and luminescence efficiency. Further increasing the calcination temperature to 800–900 °C results in partial grain coarsening and a decrease in defect density, which may adversely affect the number of traps and afterglow duration.

As shown in Figure 2d, the Rietveld refinement results show a close agreement between the experimental data and the calculated curves, with the residual curves close to zero, indicating a reliable fit (Rwp = 6.38%, Rp = 4.80%, GOF = 1.64). The composite phosphor consists of two main crystalline phases:

Ca_1.93_Sr_1.07_Al_2_O_6_ ≈ 45.2 wt%, which serves as a matrix and provides a stable crystal framework;

SrAl_2_O_4_ ≈ 54.8 wt%, which contains Eu^2+^ luminescence centers and is the primary long-lasting luminescence-contributing phase.

These two phases form a symbiotic structure within the material. The partial miscibility of Ca^2+^/Sr^2+^ ions between the two lattices effectively modulates the local crystal field environment, contributing to the optimization of the Eu^2+^ luminescence energy level and thus improving long-lasting performance.

These results demonstrate that this composite design strategy successfully combines the structural stability of (Ca,Sr)_3_Al_2_O_6_ with the strong luminescence of SrAl_2_O_4_, creating a synergistic platform for engineering interface trap states to achieve excellent long-lasting luminescence.

### 3.2. Elemental Composition Analysis

**Figure 3:**
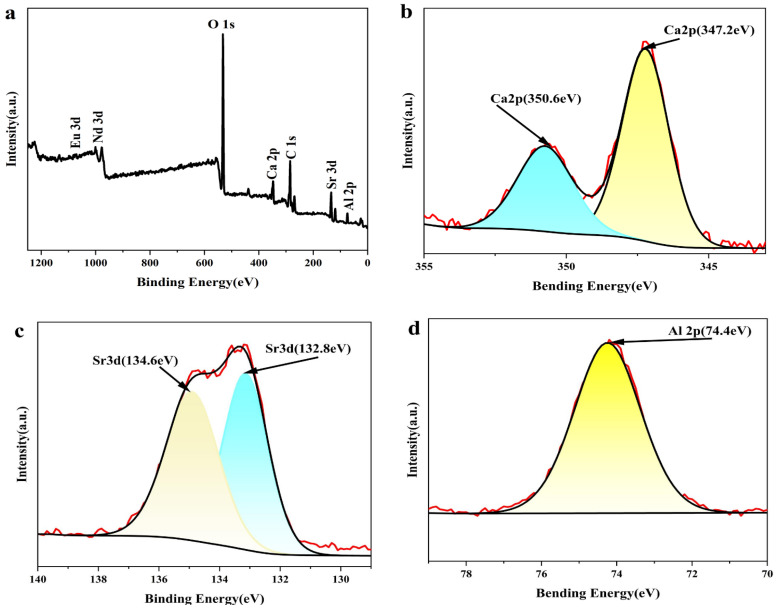

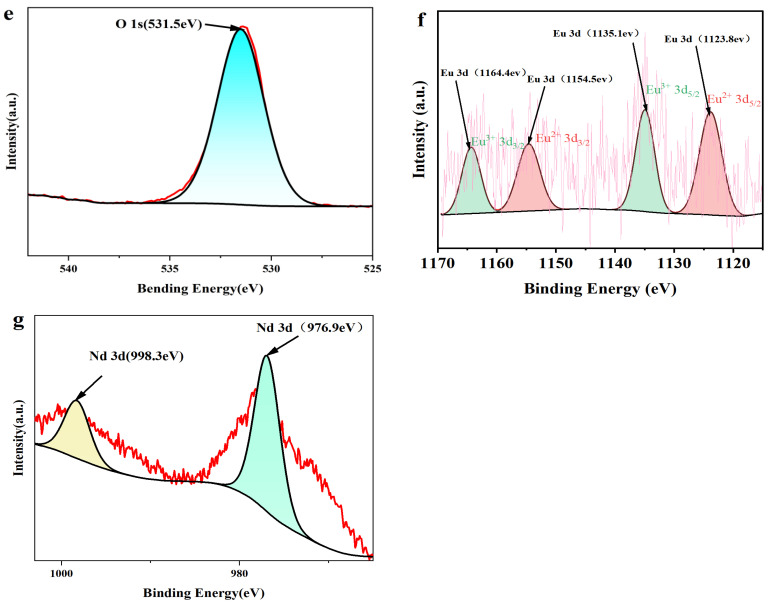
(**a**) Full XPS spectrum of the Eu^2+^, Nd^3+^-codoped (Ca,Sr)_3_Al_2_O_6_-SrAl_2_O_4_ composite long-lasting luminescent material (the primary phase is (Ca,Sr)_3_Al_2_O_6_, accompanied by a small amount of SrAl_2_O_4_ as a secondary phase); (**b**–**g**) High-resolution XPS spectra corresponding to Ca 2p, Sr 3d, Al 2p, O 1s, Eu 3d, and Nd 3d, respectively, further verifying that Eu^2+^ and Nd^3+^ successfully enter the lattice and participate in the formation of the composite structure(the red line is the original data line, and the inner black line is the fitting line).

To further confirm the valence states and chemical environments of the elements in the prepared luminescent material, X-ray photoelectron spectroscopy (XPS) analysis was performed on the (Ca,Sr)-Al-O: 0.02 Eu^2+^, 0.003 Nd^3+^ sample [24,25]. Combined with previous XRD results, the system exhibits a complex structure with (Ca,Sr)_3_Al_2_O_6_ as the primary phase and SrAl_2_O_4_ as the secondary phase. The outer red curve in Figure 3 represents experimental data, while the inner curve represents the Gaussian fit result.

As shown in Figure 3a, characteristic peaks of Ca 2p, Sr 3d, Al 2p, O 1s, Eu 3d, and Nd 3d were detected in the sample. Figure 3b shows that the binding energy of Ca 2p is located at 350.6 eV and 347.2 eV, corresponding to Ca 2p_1/2_ and Ca 2p_3/2_, indicating that Ca is in a normal divalent state. Figure 3c shows that the binding energy peaks of Sr 3d are located at 134.6 eV and 132.8 eV, corresponding to Sr 3d_3/2_ and Sr 3d_5/2_, respectively, indicating that the Sr element also maintains a stable valence state.

The high-resolution O 1s spectrum (Figure 3e) is presented to verify oxygen presence and surface cleanliness. Given the surface sensitivity of XPS, the ~531–532 eV region may include contributions from adsorbed -OH/-O-C species and slight surface re-oxidation; therefore, it is not used for quantitative assessment of bulk oxygen vacancies/traps. The Eu valence and luminescent center assignment rely on constrained Eu 3d deconvolution together with PL/PLE results.

Figure 3f shows the high-resolution Eu 3d XPS spectrum of the composite phosphor, deconvoluted into Eu^2+^ and Eu^3+^ components using a constrained fitting method (Voigt profile, Shirley background). The Eu^2+^ peaks are located at ~1123.8 eV (3d_5/2_) and ~1154.5 eV (3d_3/2_), while the Eu^3+^ peaks are located at ~1135.1 eV (3d_5/2_) and ~1164.4 eV (3d_3/2_), with a spin-orbit splitting of approximately 29–31 eV.

These results confirm the coexistence of Eu^2+^ and Eu^3+^ components in the sample. Considering that XPS is a surface-sensitive technique that only probes the top approximately 5–10 nanometers, the detected Eu^3+^ signal may be related to partial surface oxidation after sintering or exposure to air after synthesis, and therefore may not represent the bulk composition [26]. While Eu^2+^ ions are the primary luminescence centers responsible for broadband green emission and long afterglow, the detected Eu^3+^ signal primarily reflects surface effects and is expected to have limited influence on the persistent luminescence properties. Subsequent photoluminescence (PL) and afterglow results will further support this interpretation.

The spectrum of Nd (Figure 3g) shows binding energy peaks at 976.9 eV and 998.3 eV, corresponding to Nd^3+^ 3d_5/2_ and 3d_3/2_, indicating that Nd exists in a trivalent form and acts as a trap.

XPS results corroborate with XRD analysis: the primary phase of the material is (Ca,Sr)_3_Al_2_O_6_, and the secondary phase is SrAl_2_O_4_; Eu^2+^ exists stably as a luminescent center, while the synergistic effect of Nd^3+^ improves the trap distribution and afterglow properties. This synergistic effect of the composite structure provides the structural foundation for achieving excellent green long-lasting performance.

### 3.3. SEM Sintering Condition Analysis

**Figure 4:**
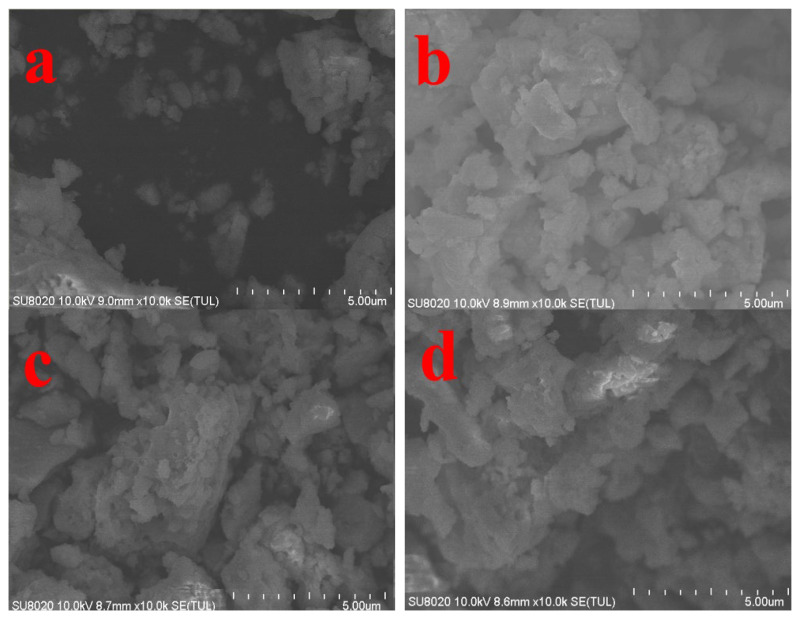

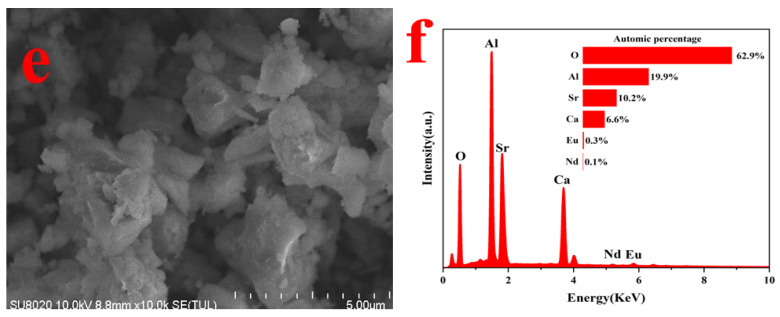
SEM images of the (Ca,Sr)_3_Al_2_O_6_-SrAl_2_O_4_: 0.02 Eu^2+^, 0.003 Nd^3+^ composite long afterglow luminescent material prepared at different calcination temperatures, in which the main phase is (Ca,Sr)_3_Al_2_O_6_, accompanied by a small amount of SrAl_2_O_4_ secondary phase: (**a**) 500 °C, (**b**) 600 °C, (**c**) 700 °C, (**d**) 800 °C, (**e**) 900 °C; (**f**) is the energy dispersive spectrum (EDS) spectrum and elemental distribution of the 700 °C sample.

Scanning electron microscopy (SEM) was used to investigate the effect of different calcination temperatures on the microstructure of the (Ca,Sr)_3_Al_2_O_6_-SrAl_2_O_4_: 0.02 Eu^2+^, 0.003 Nd^3+^ composite phase afterglow material. The results are shown in Figure 4a–e.

Overall, the particles evolve from a loose and disordered structure to a regular and dense structure with increasing temperature. At 500 °C (Figure 4a), the sample particles are small and the surface is rough, showing incompletely crystallized blocks/irregular agglomerates, which indicates that the combustion reaction is insufficient and some precursors remain.

At 600 °C (Figure 4b), the particles begin to grow, with irregular flakes and blocks coexisting, the grain boundaries are still unclear, and there are many pores.

At 700 °C (Figure 4c), the particles grow further and tend to be regularized, locally showing flakes and short columns, with a particle size concentrated at about 1–2 μm, and a significantly improved crystallinity. The (Ca,Sr)_3_Al_2_O_6_ main phase and SrAl_2_O_4_ secondary phase tend to coexist uniformly.

When the temperature continues to rise to 800 °C (Figure 4d), the particles undergo obvious sintering and agglomeration, and the grain boundaries are blurred.

At 900 °C (Figure 4e), more serious densification and particle fusion occur, and the dispersion decreases.

Overall, a temperature around 700 °C achieves an optimal balance between crystallinity and dispersion. However, excessively high temperatures lead to a decrease in specific surface area and increased agglomeration, potentially hindering the effective function of the Eu^2+^ active centers and subsequent luminescence performance.

Figure 4f shows the EDS spectrum of the 700 °C sample. The main elements detected are O (62.9%), Al (19.9%), Sr (10.2%), and Ca (6.6%). Signals for Eu (0.3%) and Nd (0.1%) were also observed, indicating that Eu^2+^ and Nd^3+^ have been successfully doped into the crystal lattice. EDS analysis combined with XRD results further confirms that the overall structure of the sample is a complex phase system consisting of a (Ca,Sr)_3_Al_2_O_6_ primary phase and a SrAl_2_O_4_ secondary phase. Notably, some particles are flaky or large, closely related to the formation of the secondary phase SrAl_2_O_4_. This secondary phase not only serves as the primary luminescent center for Eu^2+^, generating green emission at 515 nm, but also introduces defects at localized grain boundaries. The primary (Ca,Sr)_3_Al_2_O_6_ phase provides structural support and densification during sintering, and modulates trap energy levels through lattice distortion and ion migration. This synergistic effect with the secondary phase luminescent center enhances the luminescence and long-lasting performance of the Eu^2+^, Nd^3+^-doped system.

### 3.4. Luminescence Performance Analysis

**Figure 5:**
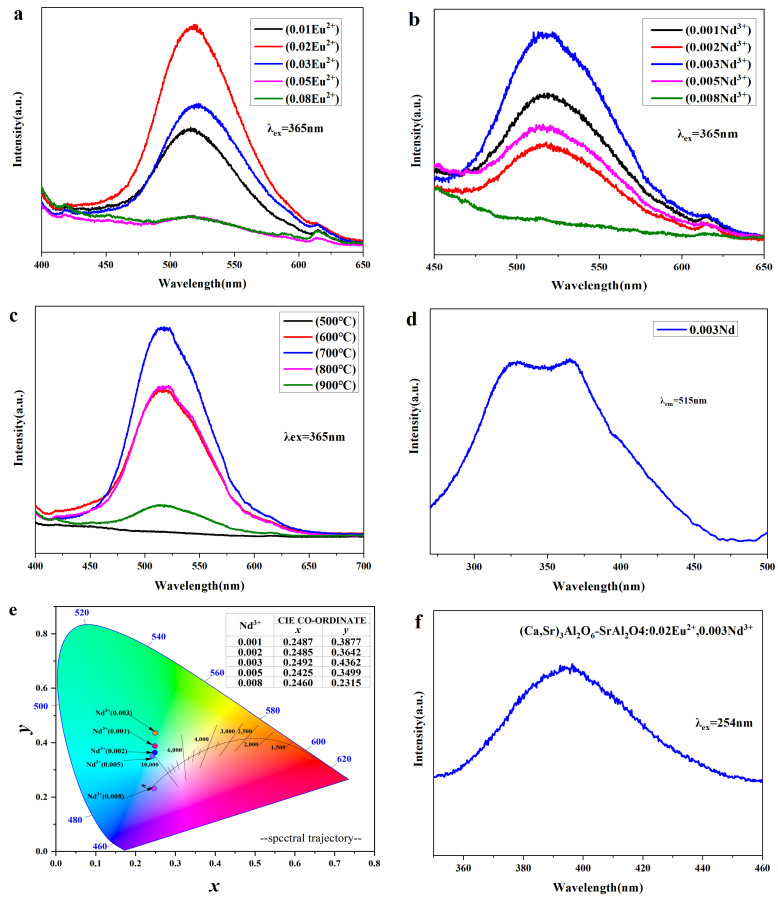

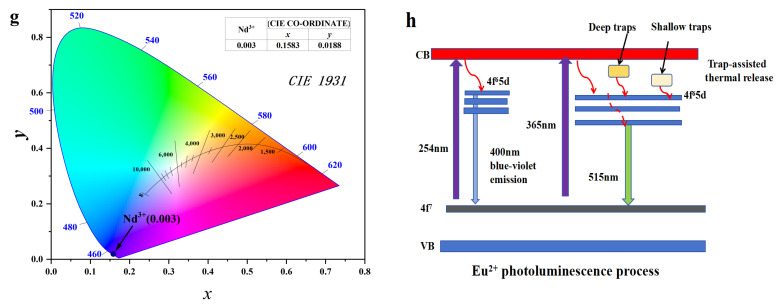
(**a**) Emission spectra of the composite luminescent material [(Ca,Sr)_3_Al_2_O_6_ primary phase with SrAl_2_O_4_ secondary phase] at different Eu^2+^ doping concentrations (x = 0–0.08) under 365 nm excitation; (**b**) Emission spectra of samples with different Nd^3+^ doping concentrations (y = 0.001–0.008) when Eu^2+^ is fixed at 0.02; (**c**) Emission spectra of samples with Eu^2+^ = 0.02 and Nd^3+^ = 0.003 at different calcination temperatures (500–900 °C); (**d**) Excitation spectra corresponding to the 515 nm emission peak; (**e**) CIE 1931 chromaticity diagram of (Ca,Sr)_3_Al_2_O_6_-SrAl_2_O_4_:0.02Eu^2+^,yNd^3+^ phosphor (y = 0.001–0.008) under 365 nm excitation. The colored dots correspond to the actual emission color positions of the samples at different Nd^3+^ concentrations, and the arrows indicate the specific positions of the chromaticity coordinates. The inset lists the corresponding CIE (x, y) coordinates. (**f**) Emission spectrum of a sample with Eu^2+^ = 0.02 and Nd^3+^ = 0.003 prepared at 700 °C under 254 nm excitation. (**g**) CIE 1931 chromaticity diagram of (Ca,Sr)_3_Al_2_O_6_-SrAl_2_O_4_: 0.02Eu^2+^,0.003Nd^3+^ phosphor under 254 nm excitation. The colored dots indicate the chromaticity positions of the samples, and the arrows highlight their precise coordinates (listed in the inset table). (**h**) Schematic diagram of the Eu^2+^ photoluminescence process. Under 254 nm or 365 nm excitation, electrons transition from the 4f^7^ ground state to the 4f^6^5d excited state, producing blue-violet (~400 nm) or green (~515 nm) emission through radiative relaxation. Under 365 nm excitation, some excited carriers are trapped in shallow and deep traps and subsequently released through thermal release, ultimately resulting in persistent luminescence.

Figure 5 shows the luminescence behavior (PL) and excitation characteristics (PLE) of composite materials composed of a (Ca,Sr)_3_Al_2_O_6_ primary phase and a SrAl_2_O_4_ secondary phase under different Eu^2+^/Nd^3+^ doping conditions and calcination temperatures at 365 nm and 254 nm excitation channels [27,28,29].

As shown in Figure 5a, under 365 nm excitation, the samples all exhibit a broadband green emission of Eu^2+^ 4f^6^5d^1^ → 4f^7^ at ≈515 nm [30,31,32]. This emission increases with the Eu^2+^ content and then decreases, reaching a peak at x(Eu^2+^) = 0.02. Further increases in concentration lead to typical concentration quenching.

Figure 5b examines the Nd^3+^ content at a fixed Eu^2+^ concentration of 0.02. Luminescence is strongest at y(Nd^3+^) = 0.003. Nd^3+^ is not a luminescent center, but rather an effective trap. A moderate amount promotes carrier capture/release and enhances afterglow, while an excess introduces competing traps and non-radiative channels, weakening luminescence [33].

In the Eu^2+^ and Nd^3+^ doped system, the emission intensity shows a non-monotonic change with concentration, first increasing and then decreasing: at low concentrations, the increase in the number of activated ions enhances luminescence; at medium concentrations, the energy transfer effect between Eu^2+^ and Nd^3+^ further improves the emission efficiency; while at high concentrations, the close distance between ions causes concentration quenching and non-radiative energy dissipation, resulting in a decrease in emission intensity.

Figure 5c demonstrates the temperature effect: the intensity first increases and then decreases with temperature, reaching its peak at 700 °C. This is consistent with the optimal ratio of crystallinity and complex phases revealed by XRD/SEM.

Figure 5d shows the excitation spectrum corresponding to the 515 nm emission. A broad absorption band is observed between 270–420 nm, with a significant response between ~350–370 nm, consistent with the 4f^7^ → 4f^6^5d^1^ transition of Eu^2+^.

Figure 5e shows the CIE 1931 chromaticity coordinates for the 365 nm channel. With increasing Nd^3+^ concentration, the chromaticity coordinates gradually shift from (*x* = 0.2492, *y* = 0.4362) to (*x* = 0.2460, *y* = 0.2315), indicating a slight increase in the relative contribution of the short-wavelength side of the green broadband, with a slight blueshift. Combined with structural characterization, it is clear that the (Ca,Sr)_3_Al_2_O_6_ primary phase modulates the radiation and energy storage/release processes of the SrAl_2_O_4_: Eu^2+^ secondary phase by altering the local lattice and defect/trap distribution. The two synergistically produce a yellow-green emission at ≈515 nm with excellent afterglow [34,35]. Overall, the optimal conditions for this system are Eu^2+^ = 0.02, Nd^3+^ = 0.003, and 700 °C.

To demonstrate the dual-mode response of the composite material, Figure 5f,g present the results for the 254 nm channel. Figure 5f (using the 700 °C sample as an example) shows a broad, smooth blue-violet band centered at approximately 400 nm, located between ≈360–460 nm. This indicates that this channel is primarily dominated by high-energy side emission from Eu^2+^ 5d → 4f, representing transient emission. Consistent with the photograph, the emission rapidly disappears after the UV light is removed, with no discernible afterglow. This result is consistent with the XPS Eu 3d deconvolution results (Figure 3f, which confirm that Eu^2+^ dominates the sample, with Eu^3+^ originating solely from surface oxidation. Furthermore, the PL spectrum under 365 nm excitation (Figure 5a) shows only broadband green emission around 515 nm, further confirming that Eu^2+^ is the primary luminescent center in this system. Figure 5g shows that the CIE 1931 coordinates for this channel lie in the deep blue-violet region (*x* ≈ 0.1583, *y* ≈ 0.0188). Considering that 254 nm excitation often produces a secondary scattering artifact at approximately 508 nm, this paper screened the 500–515 nm region during chromaticity and integral calculations to avoid shifting the color coordinates toward yellow-green.

The complex phase system exhibits green light with a long afterglow at 365 nm and a bluish-violet instantaneous emission with no afterglow at 254 nm. The readability in these two orthogonal dimensions of color and time together provides the material with a dual-mode anti-counterfeiting advantage.

The CIE chromaticity coordinates *x* and *y* can be calculated using the following formula [8]:

(1) $$x\;=\;\frac{X}{X+Y+Z}$$

(2) $$y=\frac{Y}{X+Y+Z}$$

where *X*, *Y*, and *Z* are CIE tristimulus values. The CIE 1931 *xy* chromaticity coordinates were calculated from the spectral data of samples with different Nd^3+^ ion doping concentrations, as shown in Figure 5e. It can be seen that when the Nd^3+^ ion concentration is 0.003, the color is green, with chromaticity coordinates of (*x* = 0.2492, *y* = 0.4362). Beyond 0.003, the color shifts toward blue as the concentration increases. When the doping concentration reaches 0.008, the chromaticity coordinates are (*x* = 0.2460, *y* = 0.2315), which is blue-purple as shown in Figure 5g (*x* = 0.1583, *y* = 0.0188), enhancing the dual-mode anti-counterfeiting properties.

Figure 5h proposes a Jablonski-type scheme for the (Ca,Sr)_3_Al_2_O_6_-SrAl_2_O_4_: Eu^2+^, Nd^3+^ composite. Under 365 nm excitation, electrons in Eu^2+^ are promoted from the 4f^7^ ground state to the lower crystal-field sublevels of 4f^6^5d^1^. A fraction of the excited carriers are captured by Nd^3+^-related traps located ~0.6–0.8 eV below the conduction band (CB) [36,37]. After the excitation is removed, thermally assisted de-trapping returns electrons to the Eu^2+^ 4f^6^5d^1^ state, from which radiative relaxation to 4f^7^ yields the persistent green emission at ~515 nm. Shallow traps release carriers rapidly and contribute to the early brightness, whereas deeper traps sustain the afterglow.

In contrast, 254 nm (~4.88 eV) excites carriers to high-energy states near the CB; they relax non-radiatively to the upper 4f^6^5d^1^ sublevels and then recombine radiatively to 4f^7^, giving an instantaneous blue-violet band around ~400 nm with negligible persistence. This dual-excitation picture also rationalizes the observation that the maximum prompt intensity occurs at lower Nd^3+^ (y = 0.003), while the longest afterglow appears at higher Nd^3+^ (y = 0.008): increasing Nd^3+^ raises the density/depth of traps, favoring persistence at the expense of initial intensity.

### 3.5. UV-Vis Diffuse Reflectance and Apparent Optical Onset

**Figure 6:**
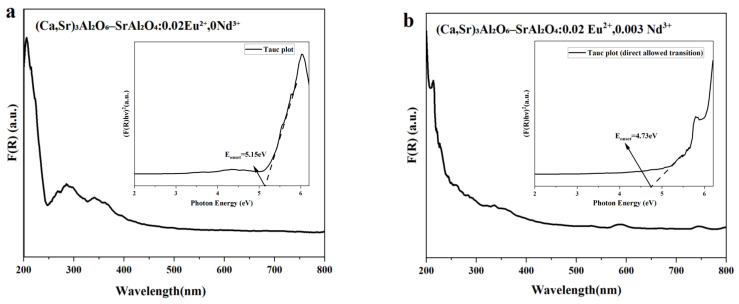
UV-Vis diffuse reflectance spectra of the composite phosphors and corresponding Tauc plots. (**a**) (Ca,Sr)_3_Al_2_O_6_-SrAl_2_O_4_:0.02Eu^2+^ without Nd^3+^; (**b**) (Ca,Sr)_3_Al_2_O_6_-SrAl_2_O_4_:0.02Eu^2+^, 0.003Nd^3+^. The insets show the Tauc plots ((F(R)hν)^2^ vs. hν), where the dashed lines represent the linear fitting used to extrapolate the absorption edge (E_onset). The intercepts with the photon energy axis give E_onset ≈ 5.15 eV for the Eu^2+^-only sample and ≈ 4.73 eV for the Eu^2+^-Nd^3+^ sample.

As shown in Figure 6a,b, all samples exhibit a broad absorption band in the range of 250–400 nm, which can be assigned to the 4f → 5d transition of Eu^2+^ ions. To evaluate the absorption edge, the diffuse reflectance spectra were transformed using the Kubelka-Munk function and analyzed with the Tauc relation: (F(R)hν)^2^∝(hν−Eonset) [34], assuming a direct-allowed transition. The linear extrapolation of the Tauc plots gives apparent optical onset values of ~5.15 eV for the Eu^2+^-only sample and ~4.73 eV for the Eu^2+^-Nd^3+^ (0.003) co-doped sample. This shift should not be interpreted as a real narrowing of the intrinsic host bandgap. Instead, it reflects enhanced sub-band-gap absorption associated with Eu^2+^ 5d states and Nd^3+^-related defect/trap levels. Therefore, the observed change in E_onset_ mainly originates from dopant- and defect-induced localized states rather than intrinsic conduction or valence band modifications.

### 3.6. Fluorescence Lifetime Test Analysis

**Figure 7:**
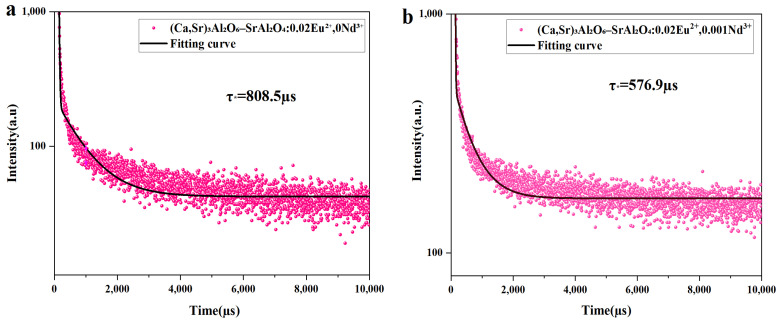

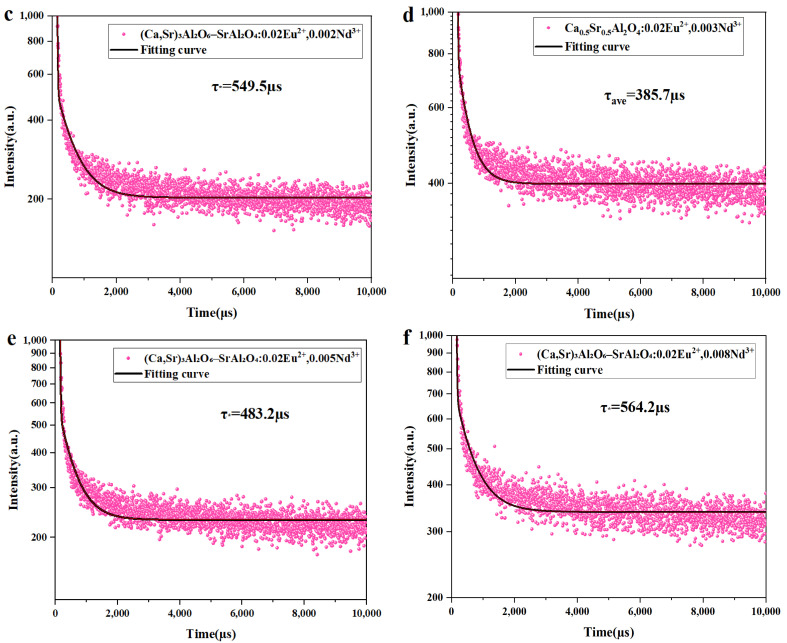
(**a**–**f**) Fluorescence decay curves and biexponential fitting results for the (Ca,Sr)_3_Al_2_O_6_-SrAl_2_O_4_: 0.02 Eu^2+^, y Nd^3+^ composite luminescent material with different Nd^3+^ doping concentrations (y = 0.001, 0.002, 0.003, 0.005, 0.008) under 365 nm excitation.

Fluorescence lifetime is the time it takes for the fluorescence intensity of a material to decay to 1/e of its initial value after the excitation light source is turned off. Figure 7a–f shows the fluorescence decay curves and biexponential fitting results of the

(Ca,Sr)_3_Al_2_O_6_-SrAl_2_O_4_: 0.02 Eu^2+^, y Nd^3+^ (y = 0.001, 0.002, 0.003, 0.005, 0.008) composite luminescent material under 365 nm excitation. The decay process typically consists of an initial rapid decay phase followed by a slow, long slow fluorescence decay component. Its variation can be fitted by the following (Equation (3) is the double exponential decay formula.) (eqs. 10.16 and 10.23 in Ref. [32]):

(3) $$I\left(t\right)\;=\;I_{0}+A_{1}e^{-\frac{t}{\tau_{1}}}+I_{0}+A_{2}e^{-\frac{t}{\tau_{2}}}$$

In the Eu^2+^-Nd^3+^ system, two parallel decay channels are common:

Different release barriers corresponding to shallow and deep traps → fast component (τ_1_) and slow component (τ_2_);

Or two Eu^2+^ luminescence environments/crystal field sites in the material → two radiative/non-radiative rates.

Thus, the observation of biexponential decay is reasonable and common.

Where I_0_ is the background constant, A_1_, and A_2_ are constants, t is the decay time, and τ_1_ and τ_2_ are the decay times of the exponential components. The above values can be calculated by Origin 2024 software(OriginLab Corporation, Northampton, MA, USA), and the specific values are shown in Table 2 The average decay time τ* under different Nd^3+^ doping concentrations can be calculated by the following (Formula (5) is the weighted average lifetime τ* calculated after fitting (eqs. 10.20 in Ref. [32]):

(4) $$\tau *\;=\;\frac{A_{1}{\tau_{1}}^{2}+A_{2}{\tau_{2}}^{2}}{A_{1}\tau_{1}+A_{2}\tau_{2}}$$

Calculation results show that the Eu^2+^-only (Ca,Sr)_3_Al_2_O_6_-SrAl_2_O_4_: 0.02 Eu^2+^ sample exhibits the longest average lifetime (~805.5 μs), indicating that the radiative transition of Eu^2+^ from 4f^6^5d^1^ to 4f^7^ occurs with minimal nonradiative interference from trap states. Upon introducing Nd^3+^, the average lifetime gradually decreases to 576.9 μs, 549.5 μs, 385.7 μs, and 483.2 μs for y = 0.001, 0.002, 0.003, and 0.005, respectively.

This behavior can be explained by a trap-assisted carrier dynamic model: Nd^3+^ introduces suitable trap levels (*E_t_* ≈ 0.6–0.8) and increases trap density (*E_t_)*, enhancing electron capture [37]). A fraction of the excited carriers are rapidly trapped, slightly reducing the prompt radiative lifetime τ*, while the thermally stimulated release of these carriers replenishes Eu^2+^ centers, leading to a prolonged macroscopic afterglow.

The afterglow decay curves of the composite phosphors were fitted using a biexponential function to evaluate the persistence behavior. The corresponding fitting parameters and average afterglow lifetimes are summarized in Table 3, confirming that the introduction of Nd^3+^ modifies both the decay constants and the weighted average lifetimes. Furthermore, the integrated afterglow intensities over different time windows are presented in Table 4, which demonstrates that shallow traps dominate the early stage (0–10 s, ~62.3%), while deeper traps associated with Nd^3+^ contribute ~15.1% in 10–30 s and ~2% beyond 100 s. This explains why the Eu^2+^-only sample exhibits the longest τ*, whereas Nd^3+^ co-doping produces stronger and longer persistent luminescence: τ*(μs-scale PL decay) is governed by direct depopulation of Eu^2+^ excited states, while the persistent afterglow (s-scale) is controlled by carrier storage and delayed release from traps [36,37].

Therefore, appropriate Nd^3+^ doping acts as an effective trap center, optimizing both the number and depth of traps to enhance persistent luminescence, whereas excessive Nd^3+^ leads to concentration quenching by increasing nonradiative losses.

The sorted data is shown in Table 2, where only the changes in fluorescence lifetime can be observed:

### 3.7. Afterglow Life Time Test

**Figure 8:**
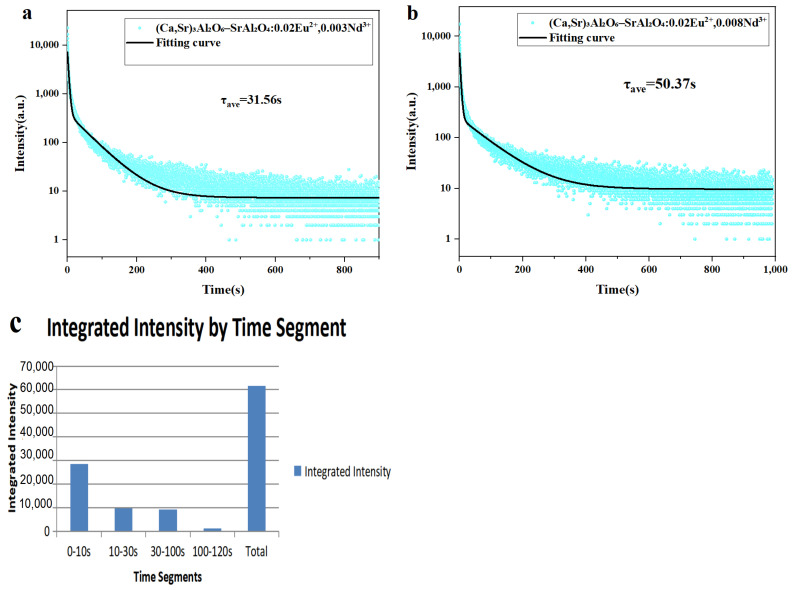
(**a**) Afterglow decay timecurve of the (Ca,Sr)_3_Al_2_O_6_-SrAl_2_O_4_: 0.02 Eu^2+^, 0.003 Nd^3+^ composite phase phosphor. (**b**) Afterglow decay curve of the (Ca,Sr)_3_Al_2_O_6_-SrAl_2_O_4_: 0.02Eu^2+^, 0.008 Nd^3+^ composite phase phosphor. (**c**) Integrated luminescence intensity of the (Ca,Sr)_3_Al_2_O_6_-SrAl_2_O_4_:0.02Eu^2+^,0.003Nd^3+^ composite-phase phosphor within different time intervals (0–10 s, 10–30 s, 30–100 s, 100–120 s) and the total integration. Note that the total value is obtained by integrating the full decay curve, which may be slightly higher than the sum of segmented parts due to baseline and tail contributions.

To further investigate the long-lasting afterglow characteristics of the (Ca,Sr)_3_Al_2_O_6_ primary phase-SrAl_2_O_4_ secondary phase composite system, afterglow decay tests were conducted on samples with varying Nd^3+^ doping concentrations [38,39], all at an excitation wavelength of 365 nm. Two representative sample groups, (Ca,Sr)_3_Al_2_O_6_-SrAl_2_O_4_: 0.02 Eu^2+^, 0.003 Nd^3+^ and (Ca,Sr)_3_Al_2_O_6_-SrAl_2_O_4_: 0.02 Eu^2+^, 0.008 Nd^3+^, were selected for comparison. The afterglow decay curves are shown in Figure 8.

The decay curves for both groups of samples fit a biexponential model, from which the average afterglow lifetime (τ_ave_) was calculated. The results show that the decay process consists of two stages: an initial rapid decay and a subsequent slow decay. The former primarily affects the initial brightness, while the latter determines the long-lasting luminescence characteristics, which is consistent with relevant research results. Figure 8a shows that the average afterglow lifetime of the sample (Ca,Sr)_3_Al_2_O_6_-SrAl_2_O_4_: 0.02 Eu^2+^, 0.003 Nd^3+^ is 31.56 s. Figure 8b shows that when the Nd^3+^ doping level increases to 0.008, τ_ave_ increases to 50.37 s. This phenomenon suggests that the introduction of Nd^3+^ creates more suitable trap levels in the lattice, improving the efficiency of carrier capture and release, thereby effectively suppressing rapid energy dissipation and prolonging afterglow.

Structural analysis reveals that the primary phase (Ca,Sr)_3_Al_2_O_6_ ensures the material’s structural stability and regulates trap distribution through local lattice distortion. The secondary phase, SrAl_2_O_4_, provides the primary emission channel for the Eu^2+^ luminescence centers. Together, these two phases, along with the participation of Nd^3+^, form a trap-emission center-lattice synergistic mechanism [40,41,42,43], contributing to the material’s excellent long-lasting luminescence performance.

The total emitted luminescence I was quantitatively evaluated by calculating the integrated intensity over the entire decay process according to the following equation [32]:

*I* = ∫*I*(*t*)*dt* (5)

where *I(t)* represents the instantaneous emission intensity at time t, and the integration is performed over the measurement period. This approach enables a more comprehensive comparison of the overall persistent luminescence performance, as it accounts for the cumulative photon output rather than only the initial brightness.

As shown in Figure 8c and Table 4, the integrated intensities of the (Ca,Sr)_3_Al_2_O_6_-SrAl_2_O_4_: 0.02 Eu^2+^, 0.003 Nd^3+^ composite phosphor were calculated for different time intervals. The results indicate that the integrated intensity within the 0–30 s range contributes approximately 62.3% of the total emission, while the emission after 30 s originates from weak but long-lasting afterglow due to carriers trapped in deeper energy levels. Therefore, the integrated intensity I provides a reliable quantitative indicator for evaluating the persistent luminescence properties.

As shown in Figure 8c and summarized in Table 4, the integrated intensity of the (Ca,Sr)_3_Al_2_O_6_-SrAl_2_O_4_: 0.02 Eu^2+^, 0.003 Nd^3+^ composite phosphor was calculated over different time intervals using trapezoidal integration. The results demonstrate that the majority of the persistent luminescence is concentrated within the first 30 s, where the integrated intensities for the 0–10 s and 10–30 s intervals reach 28,626 a.u. and 9734 a.u., respectively, contributing approximately 62.3% of the total emission.

Although the afterglow intensity decreases sharply after 30 s, the 30–100 s region still provides a non-negligible contribution of 9306 a.u. (~15.1% of the total), indicating the presence of weak but long-lasting emission. Beyond 100 s, the contribution becomes minimal (1261 a.u., <2%), suggesting that only a small fraction of carriers remain trapped in deep levels.

These results reveal a typical two-stage decay behavior: a rapid initial decay dominated by shallow trap release within the first 30 s, followed by a slow attenuation tail attributed to deep traps at longer times. Therefore, when evaluating the persistent luminescence performance, the integrated intensity within the 0–30 s range serves as the primary indicator, while the emission beyond 30 s can be regarded as a weak long-tail afterglow.

## 4. Application and Effect Testing

### 4.1. Ink Preparation and Screen Printing

After comprehensive evaluation of fluorescence intensity and afterglow duration, the sample with Eu^2+^ = 0.02 and Nd^3+^ = 0.003 exhibited the best fluorescence intensity and afterglow performance, making it a suitable material for preparing fluorescent anti-counterfeiting ink [44,45].

**Figure 9:**
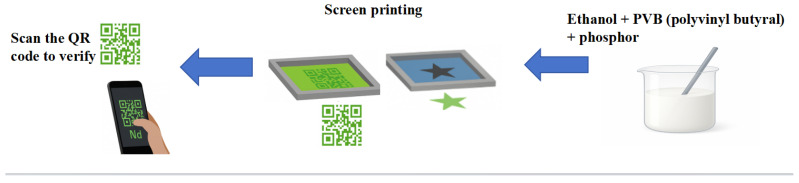
Preparation and printing process of green fluorescent anti-counterfeiting powder ink.

Ethanol and PVB (polyvinyl butyral) were mixed and the dosages adjusted to achieve a viscosity suitable for screen printing. The sample with the best luminescence performance (Eu^2+^ = 0.02, Nd^3+^ = 0.003) was selected as the luminescent matrix. The volume ratio was adjusted to 1:4, and continuous stirring was performed to ensure that the phosphor was evenly distributed in the mixed solution to prepare the anti-counterfeiting ink. PVB was chosen as the binder because of its excellent film-forming properties and compatibility with the phosphor particles. Figure 9 shows a schematic diagram of the fluorescent anti-counterfeiting ink preparation and screen printing process.

In this study, to meet the requirements of high-resolution QR code printing, we used a different formulation from previous work: an ethanol + polyvinyl butyral (PVB) system. Compared to PAA, PVB exhibits superior film-forming properties, adhesion, and mechanical stability, significantly improving the dispersion of luminescent powders and the durability of patterns.

We mixed a composite (Ca,Sr)_3_Al_2_O_6_-SrAl_2_O_4_: Eu^2+^, Nd^3+^ luminescent powder with ethanol and PVB in appropriate proportions. After stirring thoroughly, Screen printing of star and QR code patterns on coated paper. The resulting pattern was dried at 60 °C for 30 min, forming a dense and uniform luminescent layer suitable for machine-readable QR code anti-counterfeiting applications.

### 4.2. Anti-Counterfeiting Application Performance

**Figure 10:**
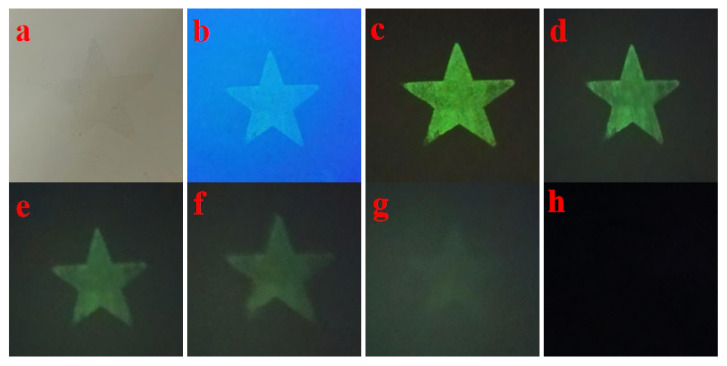
(**a**) The pattern under fluorescent light, (**b**) the pattern under 365nm UV light, and (**c**–**h**) the patterns displayed 0 s, 5 s, 10 s, 15 s, 20 s, and 35 s after the UV light is removed.

To verify the application potential of the prepared complex phase fluorescent material in anti-counterfeiting printing, this study selected the (Ca,Sr)_3_Al_2_O_6_ main phase-SrAl_2_O_4_ secondary phase: 0.02 Eu^2+^, 0.003 Nd^3+^ sample with the best performance under 700 °C firing conditions, mixed it with ethanol + PVB (polyvinyl butyral) to prepare anti-counterfeiting ink for screen printing, and printed a star pattern on the paper base, as shown in Figure 10.

As can be seen from Figure 10a, the pattern printed under fluorescent light is almost invisible, showing a good invisible effect; under 365nm ultraviolet light, as shown in Figure 10b, the star pattern is clearly visible, showing a bright green emission dominated by the Eu^2+^ luminescence center in the SrAl_2_O_4_ secondary phase. After the UV light source is turned off, as shown in Figure 10c–h, the ink still maintains a significant long-lasting afterglow, which lasts for a long time, demonstrating excellent anti-counterfeiting identification properties. The presence of the main phase (Ca, Sr)_3_Al_2_O_6_ ensures the structural stability of the system and regulates the trap distribution through lattice distortion; the secondary phase SrAl_2_O_4_ provides the Eu^2+^ green luminescence channel; and the introduction of Nd^3+^ ions forms a suitable trap energy level, effectively extending the afterglow lifetime. The synergistic effect of the three achieves the triple conversion characteristics of “invisibility-visibility-afterglow”.

**Figure 11:**
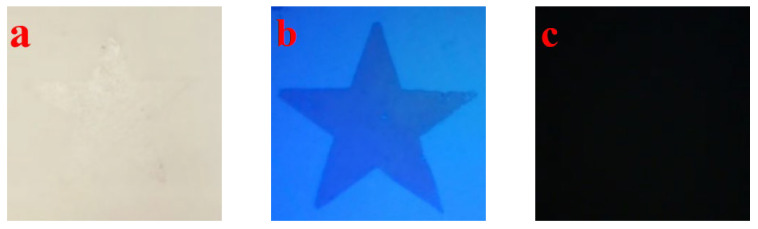
(**a**) The pattern under fluorescent light, (**b**) the pattern under 254 nm UV light, and (**c**) the display effect after the UV light is removed.

As shown in Figure 11b, under 254 nm UV excitation, the star pattern appears blue and purple instantaneous emission, corresponding to the 4f^6^5d^1^ → 4f^7^ transition of Eu^2+^, with a limited contribution from Eu^3+^ emission. After removing the UV light, no significant afterglow is observed. In contrast, under 365 nm excitation, the sample exhibits a long green afterglow of 515 nm, originating from the synergistic effect of Eu^2+^ centers and Nd^3+^ traps. This system thus achieves dual-color switchable anti-counterfeiting functionality: blue-violet prompt emission and green long afterglow.

**Figure 12:**
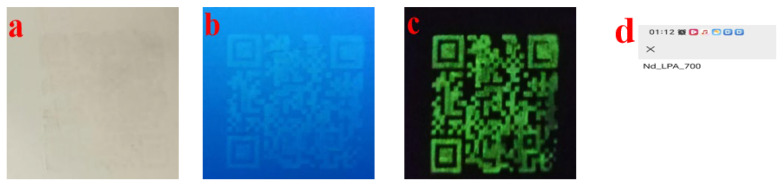
(**a**) QR code pattern under fluorescent light; (**b**) QR code pattern under 365nm UV light; (**c**) afterglow QR code pattern display effect after turning off the UV light; (**d**) anti-counterfeiting information displayed when scanning the QR codes in (**b**,**c**).

As shown in Figure 12, the anti-counterfeiting QR code printed in this study exhibits excellent visualization and machine-readable performance under different conditions. Under sunlight (Figure 12a), the QR code pattern is almost invisible, demonstrating excellent invisible anti-counterfeiting properties. Under 365 nm ultraviolet light (Figure 12b), the QR code exhibits bright green fluorescence, which can be normally identified by mobile phones or industrial machine-readable equipment. After turning off the UV light (Figure 12c), the QR code maintains a long green afterglow for >30 s and can still be scanned and verified. Figure 12d shows the code scanning interface, which returns the information “Nd_LPA_700”, indicating that the QR code corresponds one-to-one with the database information, achieving a combination of visual anti-counterfeiting and information-based traceability.

**Figure 13:**
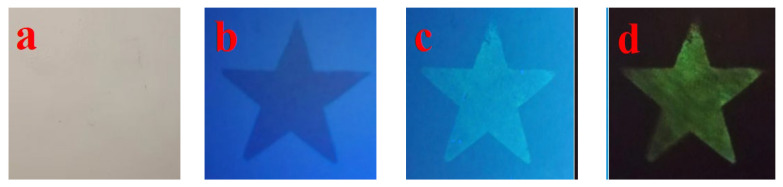

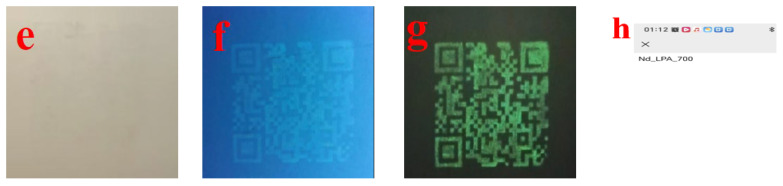
Durability of screen-printed star and QR code patterns under exposure to ambient air, two hours of continuous UV light, and accelerated stress conditions: (**a**) Star-shaped printed pattern under natural light; (**b**) Fluorescence contrast enhancement of the star pattern under 254 nm UV light excitation; (**c**) Fluorescence contrast enhancement of the star pattern under 365 nm UV light excitation; (**d**) Long afterglow after 5 s of UV light off. (**e**) QR code printed pattern under natural light; (**f**) Initial fluorescence of the QR code under 365 nm UV light excitation; (**g**) Long afterglow after 5 s of UV light off, with the QR code still readable; (**h**) Scanning the QR code with a smartphone successfully verifies the anti-counterfeiting information.

To further evaluate the practical anti-counterfeiting potential and environmental stability of the developed phosphor, Figure 13 shows the optical performance of the printed pattern after long-term environmental exposure and accelerated durability testing. After 15 days of exposure to ambient air, continuous irradiation with a 365 nm UV lamp for 2 h, wiping with a lint-free cloth 30 times, and five repeated 180° tape peeling cycles, the star pattern and QR code remained invisible under natural light (Figure 13a,e), demonstrating excellent concealment capabilities. Under 365 nm excitation (Figure 13b,f), the printed pattern exhibited bright green luminescence originating from the 4f^6^5d^1^ → 4f^7^ transition of Eu^2+^ (Figure 13c), facilitating rapid verification. A clear and persistent afterglow was still observed after the UV light source was turned off (Figure 13d,g), demonstrating excellent long-term luminescence stability even under combined environmental and mechanical stress. Importantly, the QR code can still be accurately scanned using a smartphone app [Figure 13h], demonstrating that the phosphor-based ink maintains high readability and reliable anti-counterfeiting functionality under real-world conditions.

## 5. Conclusions and Outlook

The composite long-lasting afterglow material obtained in this study consists of a primary phase of (Ca,Sr)_3_Al_2_O_6_, accompanied by a secondary phase of SrAl_2_O_4_. Through co-doping with Eu^2+^ and Nd^3+^, both ions successfully integrate into the crystal lattice, with the primary phase providing a stable crystal framework and the secondary phase serving as the primary luminescence center. Due to a certain degree of Ca^2+^/Sr^2+^ ion miscibility within the composite structure, the material exhibits tunable luminescence similar to that of a solid solution [46,47].

Eu^2+^ is more readily incorporated into the SrAl_2_O_4_ lattice, primarily due to the matching ionic radius and coordination environment: Eu^2+^ (approximately 1.25 Å, CN = 8) is nearly identical to Sr^2+^ (approximately 1.26 Å, CN = 8), but significantly different from Ca^2+^ (approximately 1.12 Å). Furthermore, the large octacoordinate radius of SrAl_2_O_4_ provides a favorable environment for the stable existence of Eu^2+^.The combined results of XPS and PL indicate that Eu^2+^ is the main luminescence center, while the smaller Eu^3+^ component mainly comes from surface oxidation. Afterglow decay and lifetime curves show that energy release follows the principle of rapid release from shallow traps and slow release from deep traps, indicating that luminescence and energy storage are primarily dependent on the SrAl_2_O_4_ phase. In contrast, (Ca,Sr)_3_Al_2_O_6_-SrAl_2_O_4_, While not directly contributing to the primary luminescence in the system, (Ca,Sr)_3_Al_2_O_6_-SrAl_2_O_4_ significantly influences the afterglow characteristics and luminescence stability by creating appropriate trap energy levels, regulating carrier capture and release behavior, and providing lattice structural support. Therefore, it plays a key role in the overall performance of the material, providing “auxiliary trapping and structural regulation.”

Under 365 nm excitation, the material produces broadband green emission at ≈515 nm. Taking emission intensity as the main optimization goal, the optimal synthesis parameters were determined by systematically optimizing the doping concentration and heat treatment conditions: Eu^2+^ = 0.02, Nd^3+^ = 0.003, and sintering temperature of 700 °C. UV-visible diffuse reflectance results show that the sub-bandgap absorption caused by doping leads to a slight shift in the apparent optical onset energy (E_onset_), which does not represent a real change in the intrinsic bandgap. Under optimized conditions, the afterglow time of the material exceeds 30 s, combining high brightness and long lifetime characteristics.

The fluorescent anti-counterfeiting ink prepared based on this composite material achieves the triple response characteristics of “invisibility-visibility-long afterglow.” The printed high-resolution QR code is nearly invisible in sunlight, but exhibits bright green emission under 365 nm ultraviolet excitation. Even after the light source is turned off, the long afterglow remains recognizable and can be accurately read by mobile phones and industrial barcode scanners. Furthermore, under 254 nm excitation, the material produces transient blue-violet emission, achieving dual-mode color response anti-counterfeiting, further improving the reliability and security of anti-counterfeiting. Compared to the traditional single-phase SrAl_2_O_4_: Eu^2+^, Nd^3+^ system, the (Ca,Sr)_3_Al_2_O_6_-SrAl_2_O_4_ multiphase trap control strategy proposed in this study not only achieves high-brightness, long-lasting green emission, but also combines the combined advantages of dual-mode excitation response and high-resolution QR code anti-counterfeiting. This achievement significantly expands the material’s application potential in high-security anti-counterfeiting, information encryption, and intelligent traceability.

## Figures and Tables

**Table 1:** Concentration of nitrate prepared.

Ca(NO_3_)_2_	Sr(NO_3_)_2_	Al(NO_3_)_3_	Eu(NO_3_)_3_	Nd(NO_3_)_3_
1 mmol/mL	0.5 mmol/mL	1 mmol/mL	0.1 mmol/mL	0.1 mmol/mL

**Table 2:** Fluorescence lifetime fitting parameters and average lifetime values for the (Ca,Sr)_3_Al_2_O_6_-SrAl_2_O_4_: 0.02 Eu^2+^, y Nd^3+^ composite luminescent material at various Nd^3+^ doping concentrations.

Fluorescence Lifetime (µs)						
Nd^3+^	0.000	0.001	0.002	0.003	0.005	0.008
A_1_	1.43	8.38	4.69	5.57	3.35	1.79
τ_1_	15.59	10.00	13.52	13.28	14.08	15.25
A_2_	187.94	388.50	392.50	546.55	423.78	445.81
τ_2_	813.99	576.90	549.26	385.86	483.45	564.21
τ*	808.5 µs	576.9 µs	549.5 µs	385.7 µs	483.2 µs	564.2 µs

**Table 3:** Afterglow lifetime fitting parameters and average afterglow lifetime data of. (Ca,Sr)_3_Al_2_O_6_-SrAl_2_O_4_: 0.02 Eu^2+^, 0.003 Nd^3+^ and (Ca,Sr)_3_Al_2_O_6_-SrAl_2_O_4_: 0.02 Eu^2+^, 0.008 Nd^3+^ composite phase phosphors.

Afterglow Lifetime (s)		
Nd^3+^	0.003	0.008
A_1_	7058.75	4472.70
τ_1_	3.42	3.43
A_2_	397.87	238.82
τ_2_	60.05	85.61
τ_ave_	31.56 s	50.37 s

**Table 4:** Statistics of the integrated luminescence of the (Ca,Sr)_3_Al_2_O_6_-SrAl_2_O_4_: 0.02 Eu^2+^, 0.003 Nd^3+^ composite phosphor at different time intervals.

Segment	0–10 s	10–30 s	30–100 s	100–120 s	Total
Integrated_Intensity	28,626.48	9733.73	9305.71	1261.16	61,498.50

## Data Availability

Data is contained within the article.
